# Based on network pharmacology and molecular docking to explore the protective effect of Epimedii Folium extract on cisplatin-induced intestinal injury in mice

**DOI:** 10.3389/fphar.2022.1040504

**Published:** 2022-10-12

**Authors:** Juan Xia, Jun-Nan Hu, Zi Wang, En-Bo Cai, Shen Ren, Ying-Ping Wang, Xiu-Juan Lei, Wei Li

**Affiliations:** ^1^ Institute of Special Animal and Plant Sciences of Chinese Academy of Agricultural Sciences, Changchun, China; ^2^ College of Life Sciences, Jilin Agricultural University, Changchun, China; ^3^ College of Chinese Medicinal Materials, Jilin Agricultural University, Changchun, China; ^4^ National and Local Joint Engineering Research Center for Ginseng Breeding and Development, Changchun, China

**Keywords:** Epimedii Folium extract, cisplatin, intestinal injury, network pharmacology, PI3K/Akt signaling pathway

## Abstract

**Background:** Epimedii Folium, as a natural botanical medicine, has been reported to have protective effects on intestinal diseases by modulating multiple signaling pathways. This study aimed to explore the potential targets and molecular mechanisms of Epimedii Folium extract (EFE) against cisplatin-induced intestinal injury through network pharmacology, molecular docking, and animal experiments.

**Methods:** Network pharmacology was used to predict potential candidate targets and related signaling pathways. Molecular docking was used to simulate the interactions between significant potential candidate targets and active components. For experimental validation, mice were intraperitoneally injected with cisplatin 20 mg/kg to establish an intestinal injury model. EFE (100, 200 mg/kg) was administered to mice by gavage for 10 days. The protective effect of EFE on intestinal injury was analyzed through biochemical index detection, histopathological staining, and western blotting.

**Results:** Network pharmacology analysis revealed that PI3K-Akt and apoptosis signaling pathways were thought to play critical roles in EFE treatment of the intestinal injury. Molecular docking results showed that the active constituents of Epimedii Folium, including Icariin, Epimedin A, Epimedin B, and Epimedin C, stably docked with the core AKT1, p53, TNF-α, and NF-κB. In verified experiments, EFE could protect the antioxidant defense system by increasing the levels of glutathione peroxidase (GSH-Px) and catalase (CAT) while reducing the content of malondialdehyde (MDA). EFE could also inhibit the expression of NF-κB and the secretion of inflammatory factors, including TNF-α, IL-1β, and IL-6, thereby relieving the inflammatory damage. Further mechanism studies confirmed that EFE had an excellent protective effect on cisplatin-induced intestinal injury by regulating PI3K-Akt, caspase, and NF-κB signaling pathways.

**Conclusion:** In summary, EFE could mitigate cisplatin-induced intestinal damage by modulating oxidative stress, inflammation, and apoptosis.

## Introduction

Cisplatin is a broad-spectrum and highly effective platinum-based antitumor drug, which is widely used in the treatment of various solid tumors such as lung cancer (Wang et al., 2021), gastric cancer, breast cancer (Vidra et al., 2022), ovarian cancer (Muhanmode et al., 2021), and bladder cancer. However, the dose-limiting toxicities of cisplatin, such as nephrotoxicity (Ma et al., 2021), ototoxicity (Tserga et al., 2020), neurotoxicity, hepatotoxicity (Abd Rashid et al., 2021), cardiotoxicity (Xu et al., 2021), and gastrointestinal toxicity (Lopez-Tofino et al., 2021), severely limit its application in clinical oncology. Among them, gastrointestinal toxicity is the most essential clinical dose-limiting side effect, which dramatically affects the antitumor efficacy of cisplatin, seriously affects the quality of life of patients, and even needs to stop chemotherapy (Blijlevens et al., 2000). Oxidative stress plays an essential role in the pathogenesis of cisplatin-induced cytotoxicity, which indicates that the application of antioxidants can effectively ameliorate cisplatin-induced toxicity (Mansour et al., 2006). For this purpose, past studies have demonstrated that various active extracts or active components obtained from herbal medicines have a significant activity in antagonizing the side effects caused by cisplatin (Awadalla et al., 2022; Gholampour et al., 2022; Pei et al., 2022). According to literature reports, flavonoids extracted from Epimedii Folium can effectively resist the toxicity caused by cisplatin (Ma et al., 2015).

Reactive oxygen species (ROS) react with biological macromolecules (including proteins, lipids, and nucleic acids), resulting in oxidative damage to cell membranes and lipid peroxidation, thus causing extensive tissue damage (Halliwell, 2006). Cisplatin treatment can increase the biochemical indicators of intestinal oxidative stress, thereby inducing epithelial apoptosis, and ultimately leading to small intestine injury (Rashid et al., 2017). In addition, the overproduction of ROS can cause inflammation by activating the transcription factor NF-κB, leading to the massive production of cell adhesion molecules, chemokines, and proinflammatory cytokines, thereby enhancing the cytotoxicity of cisplatin (Mukhopadhyay et al., 2011). Studies have shown that the inflammatory response activated by the NF-κB signaling pathway also plays a crucial role in cisplatin-induced intestinal injury (Lee et al., 2014).

Epimedii Folium, the dried aerial part of *Epimedium sagittatum* Maxim, *Epimedium koreanum* Naka, *Epimedium brevicomu* Maxim or *Epimedium pubescens* Maxim (Chinese pharmacopoeia, 2020), is a well-known Chinese herb that has been used in functional foods and complementary medicines for more than 2000 years. Epimedii Folium has the effects of tonifying the kidney, strengthening the yang, dispelling wind, and removing dampness in the theory of traditional Chinese medicine (TCM). Hence, it is applied to prevent and treat sexual dysfunction, osteoporosis, rheumatism, neurasthenia, chronic nephritis, and cardiovascular diseases. Modern pharmacological studies have proved that Epimedii Folium has a variety of pharmacological activities, including antioxidant (Zhang et al., 2013), anti-inflammatory (Saba et al., 2020), anti-osteoporotic (Indran et al., 2016), antitumor (Song et al., 2014), anti-atherosclerosis (Xiao et al., 2017), neuroprotection (Wu et al., 2017), improving sexual function (Gu et al., 2018), and enhancing immune function (Lee et al., 2017). The intestinal tract is the most important immune organ and natural barrier, which can prevent pathogens, toxins, and other harmful substances from entering the circulation through the intestine and ensure a stable environment (Groschwitz and Hogan, 2009). Epimedii Folium combined with red ginseng synergistically relieved DSS-induced colitis in mice by modulating the NF-κB and MAPK pathways and the expression of NLRP3 (Saba et al., 2020).

Studies have shown that flavonoids extracted from Epimedii Folium have been proved to have various beneficial effects on intestinal lesions. Given this, this paper used the network pharmacology to explore the potential components, putative targets, and protective mechanisms of EFE in the treatment of cisplatin-induced intestinal injury, and conducted animal experiments for preliminary confirmation. Our findings provide a theoretical basis for the protective mechanism of EFE against cisplatin-induced intestinal damage.

## Materials and methods

### Screening active compounds of Epimedii Folium

The active ingredients of Epimedii Folium have collected through the Traditional Chinese Medicine Systems Pharmacology (TCMSP, http://tcmspw.com/tcmsp.php) Database. Based on the Oral Bioavailability (OB) ≥ 30% and Drug-Like (DL) ≥ 0.18 as thresholds, the potential active compounds of Epimedii Folium were screened by ADME analysis.

### Candidate targets collection

All the possible targets against intestinal injury have retrieved from the OMIM (https://www.omim.org/), PharmGkb (https://www.pharmgkb.org), and GeneCards (https://www.genecards.org/) database. Potential target genes of Epimedii Folium for the treatment of intestinal injury were obtained through the Veeny 2.1 (https://bioinfogp.cnb.csic.es/tools/venny/) intersection. Then we used Cytoscape (v.3.8.0) software to build the active compound-target-intestinal injury network.

### Construction of protein-protein interaction network

PPI network of acquired drug targets was created using the STRING database (https://string-db.org/). Then, the PPI network results were imported into Cytoscape v.3.8.0 (www.cytoscape.org/) for network generation and further analysis. Moreover, the median of three topological parameters, “Betweenness Centrality,” “Closeness Centrality,” and “Degree”, were calculated to evaluate the topological importance of nodes in the PPI network.

### GO and KEGG pathway enrichment analysis

To explore the role of essential target genes through bioinformatics description and annotation, GO and KEGG pathway enrichment analysis on shared target genes was performed using R software with Bioconductor package under the conditions of *p* < 0.05 and Q < 0.05, and the results were plotted in the form of a bubble chart. GO enrichment analysis includes three aspects: molecular function (MF), biological process (BP), and cellular components (CC). KEGG enrichment analysis was performed to screen out the potential signaling pathways of Epimedii Folium in the treatment of intestinal injury diseases.

### Molecular docking

To validate the association between compounds and targets, molecular docking simulations were performed using AutoDockTools-1.5.6 software. Crystal structures of crucial target proteins were downloaded from the Protein Data Bank database (http://www.rcsb.org/) in PDB format. The chemical structures of Icariin, Epimedin A, Epimedin B, and Epimedin C were obtained from the PubChem database (https://pubmed.ncbi.nlm.nih.gov/). The 3D structures of active compounds were constructed and optimized by ChemBio3D Ultra 14.0.0.117 software, and their energy was minimized using the MM2 algorithm. PyMol software removed water molecules and organic compounds from receptor proteins. The target protein receptor molecules were hydrotreated and charged by AutoDockTools-1.5.6 software, and the compounds and target protein receptors were converted to PDBQT format. Finally, Auto Dock Vina software was used to verify the molecular docking of potential targets and components. Each group of molecular docking was run three times, and the ionization energy was recorded. The visualization of docking results with the best binding ability was presented using BIOVIA Discovery Studio (2019) Visualizer.

### Animal and experimental design

Forty male SPF ICR mice (6–8 weeks old, weighing 20–25 g) were provided by Beijing Hua-Fu-Kang Biotechnology Co., Ltd., license No. SCXK (Beijing) 2019-0008. Mice were allowed free access to food and water in a rearing chamber free of specific pathogens and a 12-h light/dark cycle. The temperature controlled at 22.0°C ± 2.0°C, and the humidity maintained at 60.0% ± 10.0%.

After 1 week of acclimatization, mice were randomly allocated into 4 groups (*n* = 10): normal group, model group, cisplatin + EFE (100 mg/kg) group and cisplatin + EFE (200 mg/kg) group. Since there is currently no therapeutic agent for cisplatin-induced myocardial injury in the clinic, a group of positive drugs was not set up in the present work. EFE was dissolved in 0.05% carboxymethylcellulose sodium (CMC-Na) and administered to mice by gavage for ten consecutive days. On the 7th day of EFE administration, except the normal group, mice in the other groups were intraperitoneally injected with cisplatin 20 mg/kg to establish an acute intestinal injury model (Figure 3C).

### Ethical statement

All animal experiments were conducted according to the Guidelines for the Management and Use of Experimental Animals and were approved by the Experimental Animal Ethics Committee of Jilin Agricultural University (Animal Experiment Ethics No. 20190905002).

### Sampling

Epimedii Folium was obtained from Bozhou traditional Chinese medicine trading center (Anhui province, China), and identified as *Epimedium brevicomu* Maxim by Professor Han Mei from the College of Chinese Medicinal Materials, Jilin Agricultural University. Epimedii Folium extract was prepared and quantified according to previous reports (Zhou et al., 2019). High-performance liquid chromatography (Waters HPLC, Milford, MA) was used to quantify EFE at 317 nm on a Hypersil ODS2 column (Figures 3A,B). The quantitative analysis of the main flavonoids in EFE was as follows: 0.61% of Epimedin A, 0.87% of Epimedin B, 2.82% of Epimedin C, and 1.84% of Icariin.

### Reagents

The use of cisplatin in chemotherapy often leads to severe intestinal toxicity. Our research group has fully proved that cisplatin can cause intestinal toxicity in the body at a dose of 20 mg/kg in previous studies (Hu et al., 2021). Cisplatin (purity ≥ 99.0%) was provided by Shanghai Civic Chemical Technology Co., Ltd., (Shanghai, China); Glutathione peroxidase (GSH-Px), catalase (CAT) and malondialdehyde (MDA) detection kits and hematoxylin-eosin (H&E) staining kits were bought from Nanjing Jiancheng Bioengineering Institute (Nanjing, China); Tumor necrosis factor-α (TNF-α), interleukin-1β (IL-1β), interleukin-6 (IL-6) and diamine oxidase (DAO) enzyme-linked immunoassay (ELISA) kits were obtained from R&D Systems of the United States (Minneapolis, MN, United States); Hoechst 33258 staining kits and BCA protein concentration detection kits were acquired from Shanghai Beyotime Biotechnology Co., Ltd. (Shanghai, China); Cy3-SABC immunofluorescence staining kit was provided by BOSTER Biological Technology Co., Ltd. (Wuhan, China); Monoclonal antibodies: p-PI3K, PI3K, p-Akt, Akt, p-NF-κB, NF-κB, p-p53, p53, Bax, Bcl-2, cytochrome c, cleaved caspase-9, caspase-9, cleaved caspase-3, caspase-3, β-actin, and secondary antibodies were provided by Cell Signaling Technology (Danvers, MA, United States); Acetonitrile was chromatographic pure and obtained from Thermo Fisher Scientific Co., Ltd. (MERCK, Germany); Methanol, ethanol, and other chemical reagents were analytical pure and provided by Sinopharm Chemical Reagent Co., Ltd., (Shanghai, China).

### Determination of serum biochemical indicators

Blood samples were collected from mouse ocular venous plexus and centrifuged at 3,500 rpm for 10 min to separate serum samples. The levels of DAO, TNF-α, IL-1β, and IL-6 in serum samples were determined using ELISA kits according to the manufacturer’s instructions. 10 µl serum samples were added to 96 well plates coated with matrix and incubated at 37°C for 30 min, then chromogenic agent was added, and the absorbance of samples in each group at 450 nm was measured within 15 min. Finally, the content of biochemical indicators was calculated through the concentration-absorbance curve.

### Determination of biochemical indexes of tissue homogenate

Tissue samples were accurately weighed, 0.9% sterile saline was added according to weight (g): volume (ml) = 1:9, then homogenized with a tissue homogenizer, and finally centrifuged at 3,500 rpm for 10 min to collect the supernatant for subsequent biochemical parameter detection. The levels of MDA, GSH-Px, and CAT in tissue homogenate were determined using corresponding commercially available detection kits according to the instructions provided by Nanjing Jiancheng Bioengineering Institute.

### Histopathological examination

2∼3 cm fresh mouse duodenum, jejunum, and ileum were immersed in 10% neutral buffered formalin for at least 48 h, routinely dehydrated and deparaffinized, embedded in paraffin, and then cut into 5 µm thick sections. Paraffin sections were stained with H&E solution, and then pathological changes were observed with a light microscope, and images were collected. The degree of intestinal injury was assessed according to the histopathological scoring system.

### Hoechst 33258 staining analysis

5 μm thick tissue sections were stained with Hoechst 33258 staining solution according to the kit instructions. After standing for 5 min, the sections were washed twice with phosphate buffer (0.01 M, pH7.4) for 3 min each time and then sealed with anti-fluorescence quenching sealant. Finally, nuclear apoptosis was observed and photographed under a fluorescence microscope (Olympus BX-60, Tokyo, Japan). Hoechst 33258 staining results were quantified by Image-Pro Plus 6.0 software.

### Immunofluorometric analysis

Paraffin tissue sections were deparaffinized and hydrated with xylene and gradient ethanol solution and then repaired with citrate buffer (0.01 M, pH 6.0) under microwave conditions at medium-high temperature for 8 min. After returning to room temperature, 5% bovine serum albumin (BSA) was added dropwise to block the sections for 20 min, and then NF-κB P65 (1:100) primary antibody solution was added and incubated overnight at 4°C. Biotinylated goat anti-rabbit IgG solution was added and incubated at 37°C for 30 min. Diluted Cy3-SABC (1:400) solution was added dropwise to the sections and incubated at 37°C in the dark for 30 min. 4, 6-diamino-2-phenylindole (DAPI) solution was added for nuclear staining. Finally, the slices were sealed with anti-fluorescence quenching sealant. The fluorescence expression intensity of the antibody was observed under a fluorescence microscope (Leica TCS SP8, Germany) and photographed. Immunofluorescence quantitative analysis was performed using Image-Pro Plus 6.0 software.

### Western blot analysis

Total protein samples were obtained by lysing mouse intestinal tissues with RIPA lysis buffer supplemented with protein phosphatase inhibitors. Total protein concentration in the tissues was determined using the BCA protein quantification kit according to the instructions provided by the manufacturer. The protein samples were separated on 15% SDS-PAGE gels and transferred to polyvinylidene fluoride (PVDF) membranes by electrophoresis. Then, the PVDF membranes were sealed with 5% skimmed milk at room temperature for at least 2 h and incubated with p-PI3K (1:1,000), PI3K (1:1,000), p-Akt (1:1,000), Akt (1:1,000), p-p53 (1:1,000), p53 (1:1,000), Bax (1:2000), Bcl-2 (1:2000), cleaved caspase-9 (1:1,000), caspase-9 (1:1,000), cleaved caspase-3 (1:1,000), caspase-3 (1:1,000), cytochrome c (1:1,000), and β-actin (1:2000) primary antibody solutions at 4°C overnight. The membranes were then incubated with the HRP-conjugated secondary antibody solution at room temperature for 1.5–2 h. Finally, the intensities of protein bands were quantified by Quantity One software (Bio-Rad Laboratories, Hercules, CA, United States).

### Statistical analysis

All experimental data were expressed as mean ± standard deviation (Mean ± S.D). Statistical significance was analyzed by SPSS version 19.0 software. The statistical histograms were made by GraphPad Prism 8.04 software (GraphPad Software, La Jolla, California, United States). In all cases, *p* < 0.05 or *p* < 0.01 was considered statistically significant.

## Results

### Active compounds in Epimedii Folium and candidate targets

**FIGURE 1:**
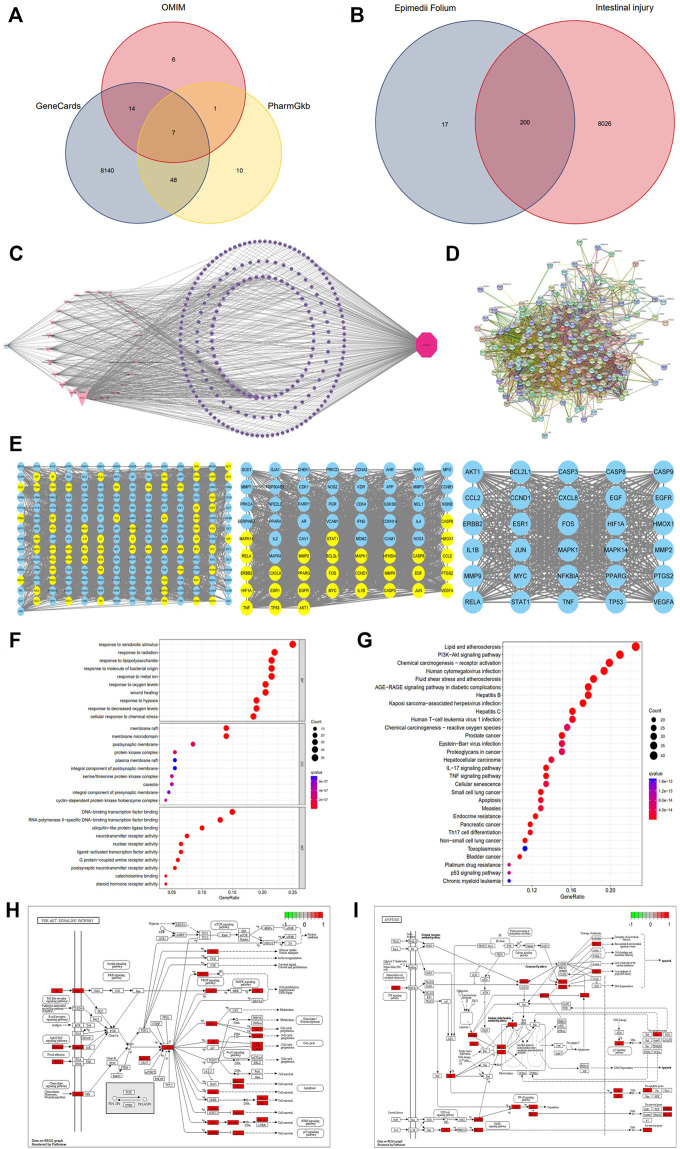
Venn diagram of three databases of intestinal injury **(A)**; Venny results of the potential target genes of Epimedii Folium in the treatment of intestinal injury **(B)**; Compound-target-disease interaction network of Epimedii Folium and intestinal injury **(C)**; The PPI network of disease-drug targets **(D)**; Network diagram of the core targets of Epimedii Folium against intestinal injury **(E)**; GO and KEGG enrichment analysis **(F–G)**; The PI3K/Akt and apoptosis signaling pathways of potential target genes of EFE in intestinal injury **(H,I)**.

Using the keyword “Epimedii Folium,” 23 active compounds were retrieved from TCMSP database. The potential active ingredients were collected under the screening conditions of OB ≥ 30% and DL ≥ 0.18. We searched through TCMSP, combined with Chinese and foreign literature supplements, deleted the active ingredients without targets, and finally obtained 25 active ingredients of Epimedii Folium, as shown in Table 1. In addition, 217 target genes were screened from the TCMSP and Swiss target prediction databases for active components. Similarly, 8,226 target genes for intestinal injury were obtained from the GeneCards, OMIM, and PharmGkb databases (Figure 1A). To obtain the targets of Epimedii Folium against intestinal damage, the co-relative targets were identified using the online Draw Venn Diagram facility. Finally, 200 overlapping targets were obtained as candidate targets of Epimedii Folium in the treatment of intestinal injury (Figure 1B). Then, the candidate targets and corresponding active compounds were imported into Cytoscape 3.7.2 software to construct the “compound-target-intestinal injury” network diagram. As shown in Figure 1C, the network diagram contained 227 nodes (25 active compounds, 200 target genes, 1 drug, and 1 disease) and 686 edges, and purple circles represent the target genes, and light pink V shapes represent the active compounds, showing the direct relationship network of active compounds with intestinal injury and Epimedii Folium.

**TABLE 1:** Candidate active components of Epimedii Folium.

Mol ID	Molecule name	OB (%)	DL
MOL001510	24-epicampesterol	37.58	0.71
MOL001645	Linoleyl acetate	42.1	0.2
MOL001771	poriferast-5-en-3beta-ol	36.91	0.75
MOL001792	DFV	32.76	0.18
MOL003044	Chryseriol	35.85	0.27
MOL003542	8-Isopentenyl-kaempferol	38.04	0.39
MOL000359	Sitosterol	36.91	0.75
MOL000422	Kaempferol	41.88	0.24
MOL004367	Olivil	62.23	0.41
MOL004373	Anhydroicaritin	45.41	0.44
MOL004380	C-Homoerythrinan,1,6-didehydro-3,15,16-trimethoxy-, (3.beta.)-	39.14	0.49
MOL004382	Yinyanghuo A	56.96	0.77
MOL004384	Yinyanghuo C	45.67	0.5
MOL004386	Yinyanghuo E	51.63	0.55
MOL004388	6-hydroxy-11,12-dimethoxy-2,2-dimethyl-1,8-dioxo-2,3,4,8-tetrahydro-1H-isochromeno [3,4-h] isoquinolin-2-ium	60.64	0.66
MOL004391	8-(3-methylbut-2-enyl)-2-phenyl-chromone	48.54	0.25
MOL004396	1,2-bis (4-hydroxy-3-methoxyphenyl) propan-1,3-diol	52.31	0.22
MOL004425	Icariin	41.58	0.61
MOL004427	Icariside A7	31.91	0.86
MOL000006	Luteolin	36.16	0.25
MOL000622	Magnograndiolide	63.71	0.19
MOL000098	Quercetin	46.43	0.28
MOL008865	Epimedin A	5.06	0.12
MOL004407	Epimedin B	8.65	0.13
MOL004409	Epimedin C	16.29	0.14

### Protein-protein interaction network analysis

The identifiable candidate targets of Epimedii Folium associated with intestinal injury were introduced into the STRING database to set up the PPI network diagram. Subsequently, the PPI network of candidate targets was inputted into Cytoscape-v3.8.0 software for visualization. We excluded disconnected nodes from the PPI network and finally obtained 197 nodes and 3,491 edges (Figure 1D). The network nodes delineate target proteins, and the edges represent protein-protein relationships. AKT1, TP53, TNF, RELA, CASP3, and CASP9 contained in the hub PPI network were considered as core targets (Figure 1E).

### GO and KEGG pathway enrichment analysis

To explore the biological functions and relevant pathways of Epimedii Folium associated with intestinal injury, GO and KEGG pathway enrichment analysis of the 200 candidate targets was carried out after calculation with *R* software. A total of 2768 GO terms (*p* < 0.05) were obtained from GO enrichment analysis, including 2,437 biological process (BP) terms, 104 cellular components (CC) terms, and 227 molecular function (MF) terms. The top 10 markedly enriched biological processes of the 200 core targets were depicted in Figure 1F, including cellular response to chemical stress, response to oxidative stress, serine/threonine protein kinase complex, and DNA-binding transcription factor binding. Moreover, 170 signaling pathways were identified by the KEGG pathway enrichment analysis (*p* < 0.05), and the finally sorted-out top 30 vital signaling pathways are shown in Figure 1G. The KEGG pathway enrichment analysis indicated that the mechanisms of Epimedii Folium against intestinal injury include the PI3K-Akt signaling pathway, apoptosis signaling pathway, p53 signaling pathway, and TNF-α signaling pathway. Additionally, the PI3K-Akt (hsa04151) and apoptosis (hsa04210) signaling pathways will be analyzed as critical pathways (Figures 1H,I).

### Molecular docking validation of core targets and active compounds

**FIGURE 2:**
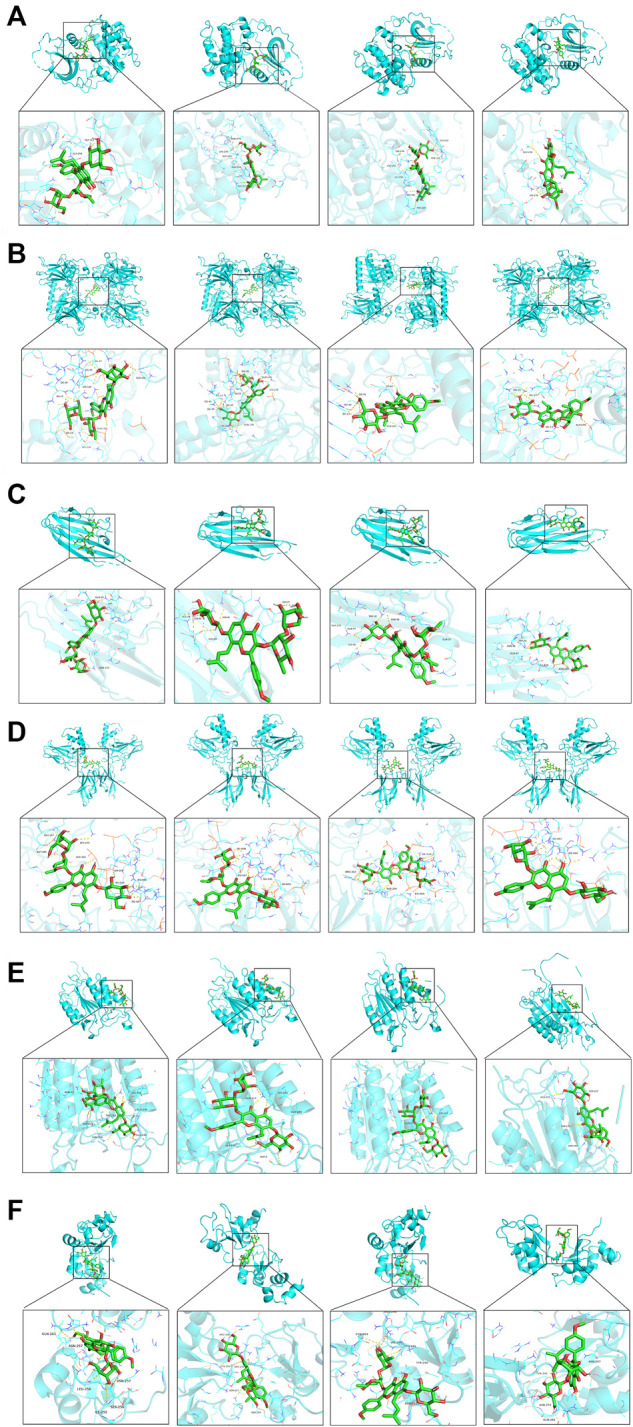
Analysis of target-compound docking simulation. **(A–F)** Epimedin A, Epimedin B, Epimedin C, and Icariin act on AKT1, P53, TNF-α, NF-κB, caspase3, and caspase9, respectively.

The PPI network screened out the core targets of Epimedii Folium against intestinal injury. To verify the reliability of candidate targets in the PPI network, four active compounds from Epimedii Folium were docked with AKT1, p53, TNF-α, NF-κB caspase 3, and caspase 9 using Auto Dock Vina software. The Vina score (kcal/mol) represented the binding affinity between the target protein and compounds. The lower the Vina score, the more stable the ligand binding to the receptor, and the greater the possibility of molecular interaction. As the score for molecular docking, the binding energy less than -5 kcal/mol indicates a more vigorous binding activity. Interestingly, the results showed that Icariin, Epimedin A, Epimedin B, and Epimedin C readily bind to AKT1, p53, TNF-α, NF-κB, caspase3, and caspase9 due to their very low binding energies (Table 2). These results provide a basis for explaining the potential of AKT1, p53, TNF-α, NF-κB, caspase 3, and caspase 9 as the key therapeutic targets for intestinal injury. Finally, 3D maps of Icariin, Epimedin A, Epimedin B, and Epimedin C binding to AKT1, p53, TNF-α, NF-κB, caspase 3, and caspase 9 are shown in Figures 2A–F.

**TABLE 2:** Molecular docking results of core targets and active ingredients.

Protein name	Molecule name	PDB	Vina scores (kcal/mol)	Mean	S	RMSD	Center		
							x	y	z
			−8.8						
	Epimedin A		−8.9	−8.83	0.058	0	−26.571	2.775	16.251
AKT1		4GV1	−8.8						
			−8.7						
	Epimedin B		−8.8	−8.7	0.1	0	−26.571	2.775	16.251
			−8.6						
			−9.1						
	Epimedin C		−9.0	−9.13	0.15	0	−26.571	2.775	16.251
			−9.3						
			−9.0						
	Icariin		−9.1	−9.1	0.1	0	−26.571	2.775	16.251
			−9.2						
			−9.5						
	Epimedin A		−9.4	−9.47	0.058	0	−40.261	27.583	−12.781
			−9.5						
			−9.4						
	Epimedin B		−9.5	−9.5	0.1	0	−40.261	27.583	−12.781
P53		3Q05	−9.6						
			−9.4						
	Epimedin C		−9.2	−9.4	0.2	0	−40.261	27.583	−12.781
			−9.6						
			−9.3						
	Icariin		−9.2	-9.3	0.1	0	-40.261	27.583	-12.781
			−9.4						
			−6.5						
	Epimedin A		−6.5	−6.53	0.058	0	45.301	52.93	13.768
			−6.6						
			−6.6						
	Epimedin B		−6.7	−6.6	0.1	0	45.301	52.93	13.768
TNF-α		5UUI	−6.5						
			−6.6						
	Epimedin C		−6.5	−6.63	0.15	0	45.301	52.93	13.768
			−6.8						
			−6.6						
	Icariin		−6.5	−6.57	0.058	0	45.301	52.93	13.768
			−6.6						
			−7.7						
	Epimedin A		−7.6	−7.7	0.1	0	16.769	61.416	0.667
			−7.8						
			−8.6						
	Epimedin B		−8.7	−8.63	0.058	0	16.769	61.416	0.667
NF-κB		1A3Q	−8.6						
			−8.8						
	Epimedin C		−8.7	−8.8	0.1	0	16.769	61.416	0.667
			−8.9						
			−8.4						
	Icariin		−8.3	−8.43	0.15	0	16.769	61.416	0.667
			−8.6						
			−6.1						
	Epimedin A		−6.0	−6.1	0.1	0	27.395	19.401	58.596
			−6.2						
			−6.6						
	Epimedin B		−6.5	−6.57	0.058	0	27.395	19.401	58.596
Caspase3		2J30	−6.6						
			−6.8						
	Epimedin C		−6.7	−6.8	0.1	0	27.395	19.401	58.596
			−6.9						
			−6.0						
	Icariin		−5.8	−6.0	0.2	0	27.395	19.401	58.596
			−6.2						
			−8.2						
	Epimedin A		−8.0	−8.1	0.1	0	6.844	−11.718	−23.923
			−8.1						
			−7.8						
	Epimedin B		−7.7	−7.83	0.15	0	6.844	−11.718	−23.923
Caspase9		3D9T	−8.0						
			−7.6						
	Epimedin C		−7.5	−7.57	0.058	0	6.844	−11.718	−23.923
			−7.6						
			−7.6						
	Icariin		−7.6	−7.63	0.058	0	6.844	−11.718	−23.923
			−7.7						

### Epimedii Folium extract improved cisplatin-induced intestinal injury

**FIGURE 3:**
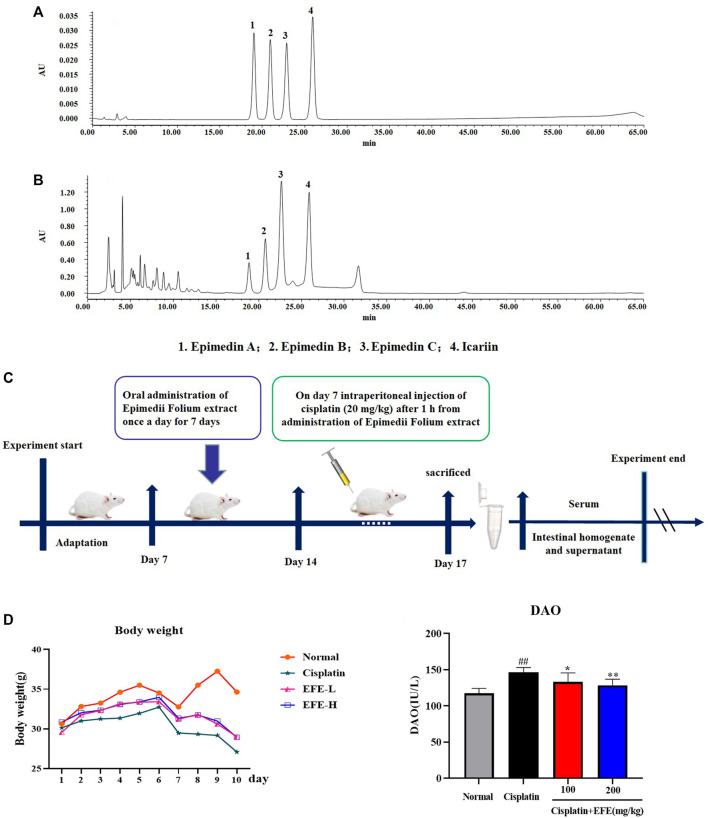
HPLC analysis of the principal components of EFE **(A–B)**; Flow chart of animal experiment design **(C)**; Effect of EFE on the weight change of mice with cisplatin-induced intestinal injury **(D)**; Effect of EFE on DAO **(E)** levels in mice. Note: Values are expressed as Mean ± S.D., (*n* = 10). ^##^ *p* < 0.01 vs. normal group; *p < 0.05, **p < 0.01 vs. model group.

As can be seen from the line chart in Figure 3D that the weights of mice in the model group decreased significantly after intraperitoneal injection of cisplatin. EFE treatment (100 and 200 mg/kg) for 10 days effectively prevented weight loss in mice with no significant difference between the two-dose groups. As shown in Figure 3E, after intraperitoneal injection of cisplatin, the level of DAO was significantly increased, which was substantially different from that in the normal group (*p* < 0.01). However, EFE administration could dramatically inhibit the increase of the biochemical index (*p* < 0.05 or *p* < 0.01), indicating that EFE could effectively alleviate the intestinal damage caused by cisplatin.

**FIGURE 4:**
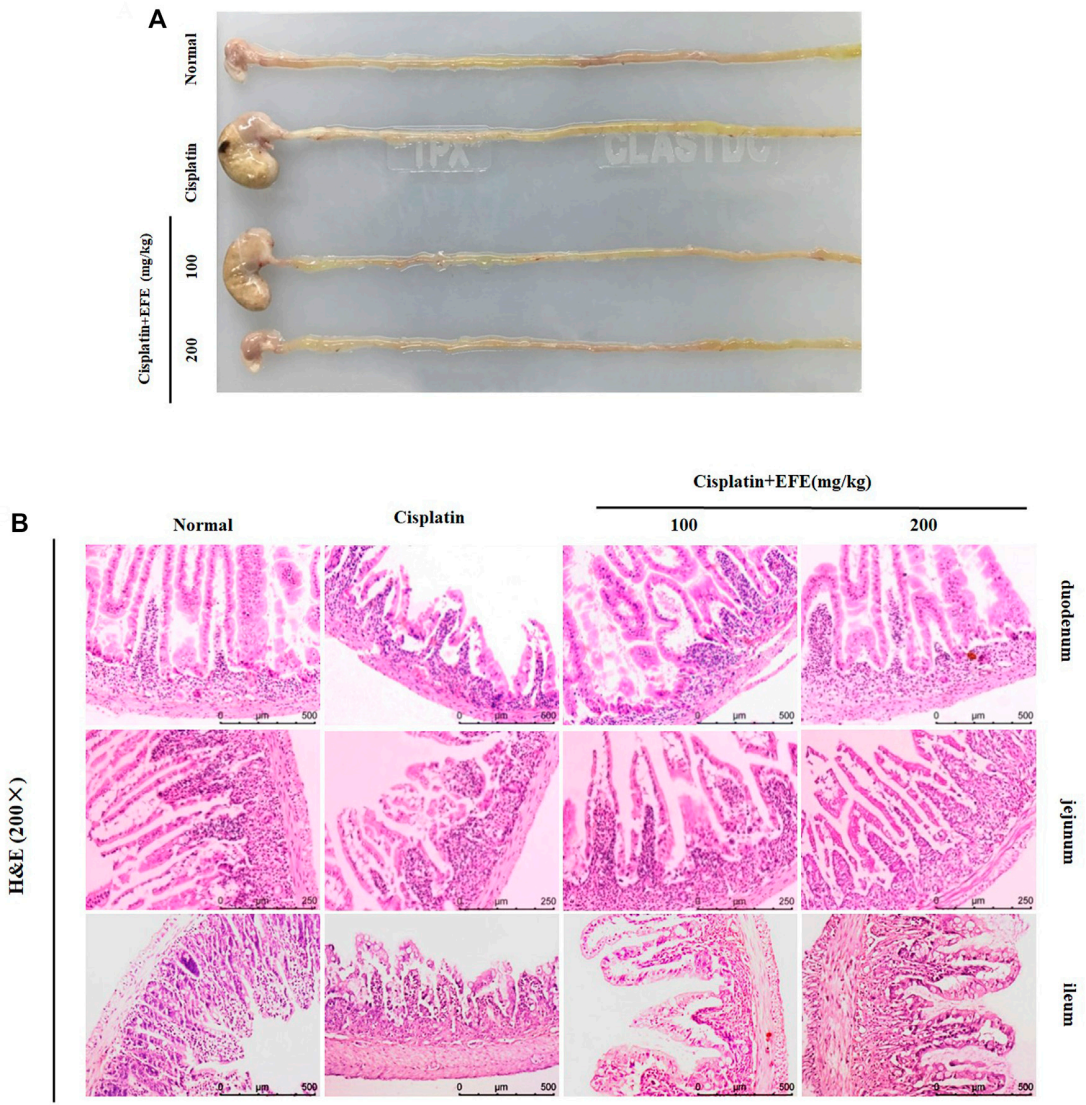
Effect of EFE on the gastrointestinal tissue morphology **(A)** of mice; Effect of EFE on intestinal histopathology in mice **(B)**. Note: Values are expressed as Mean ± S.D., (*n* = 10). ^##^ *p* < 0.01 vs. normal group; *p < 0.05, **p < 0.01 vs. model group.

Gastrointestinal Morphology of mice in each experimental group was observed (Figure 4A). It was found that the stomach of cisplatin-treated mice became larger, the color of the intestine turned white, the intestinal wall became thinner, and multiple vacuoles appeared. EFE administration could significantly improve these pathological symptoms. Histopathological observation showed that the intestinal mucosa of mice in the cisplatin group was seriously damaged, villi degeneration and desquamation, mucosal glandular structure distortion, crypt ablation, and inflammatory cell infiltration. EFE could effectively alleviate cisplatin-induced intestinal injury and significantly restore the intestinal mucosal structure (Figure 4B). Furthermore, the histomorphology and pathological sections of the mouse intestines (duodenum, jejunum, and ileum) showed that cisplatin had the most apparent damage to the duodenum. Therefore, the duodenum was selected as the research object of cisplatin-induced intestinal injury.

### Epimedii Folium extract alleviated cisplatin-induced inflammation and oxidative stress damage

**FIGURE 5:**
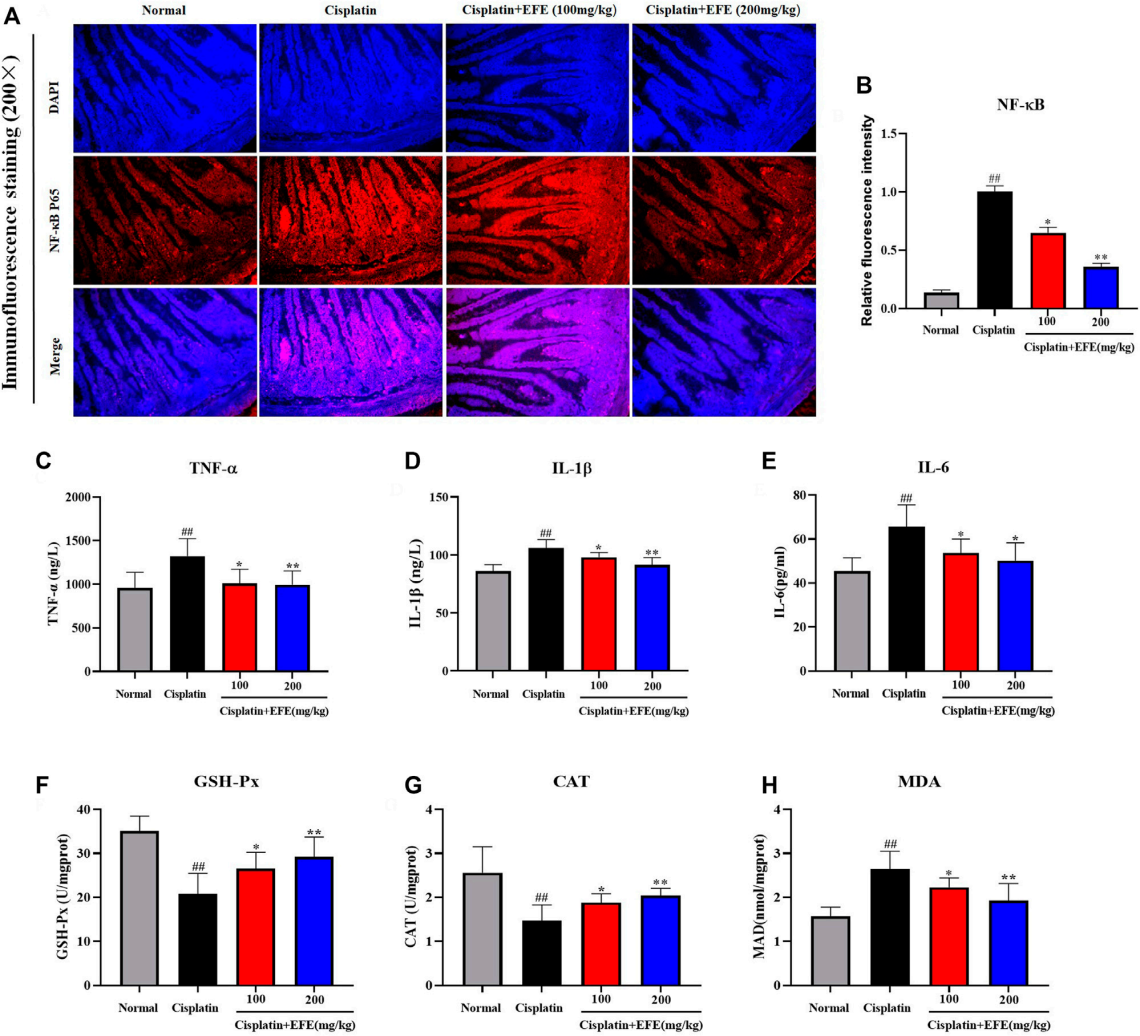
Effect of EFE on NF-κB expression in mouse intestinal tissue **(A)** and quantitative analysis of fluorescence intensity **(B)**; Effect of EFE on serum TNF-α, IL-1β, and IL-6 levels in mice **(C–E)**; Effect of EFE on GSH-Px, CAT and MDA levels in mouse intestinal tissues **(F–H)**. Note: Values are expressed as Mean ± S.D., (*n* = 10). ^##^ *p* < 0.01 vs. normal group; *p < 0.05, **p < 0.01 vs. model group.

To verify whether EFE could ameliorate cisplatin-induced intestinal injury through anti-inflammatory effects, the expression levels of NF-κB p65 in mouse intestinal tissues were detected by immunofluorescence staining. As shown in Figure 5A, NF-κB p65 had almost no fluorescence expression in the intestinal tissues of normal mice but was highly expressed in cisplatin-treated mice (*p* < 0.01). Interestingly, the intensity of fluorescence expression was significantly attenuated after EFE administration (Figure 5B) (*p* < 0.05 or *p* < 0.01). Moreover, as depicted in Figures 5C–E, the results showed that the serum levels of TNF-α, IL-1β, and IL-6 in the model group were significantly higher than those in the normal group (*p* < 0.01). However, the secretion levels of proinflammatory factors were suppressed considerably after EFE administration, especially in the high-dose EFE group (*p* < 0.05 or *p* < 0.01). These results suggested that EFE might effectively ameliorate cisplatin-induced intestinal injury through anti-inflammatory effects.

To evaluate whether EFE could protect intestinal tissues from oxidative stress damage caused by cisplatin, the levels of MDA, GSH-Px, and CAT in intestinal tissues were detected using corresponding kits (Figures 5F–H). The results showed that the levels of GSH-Px and CAT in the model group were significantly decreased, and the level of MDA was increased considerably, which was substantially different from those in the normal group (*p* < 0.01). Interestingly, EFE could reverse the changes of these oxidative stress indices (*p* < 0.05 or *p* < 0.01).

### Epimedii Folium extract inhibited cisplatin-induced intestinal apoptosis

**FIGURE 6:**
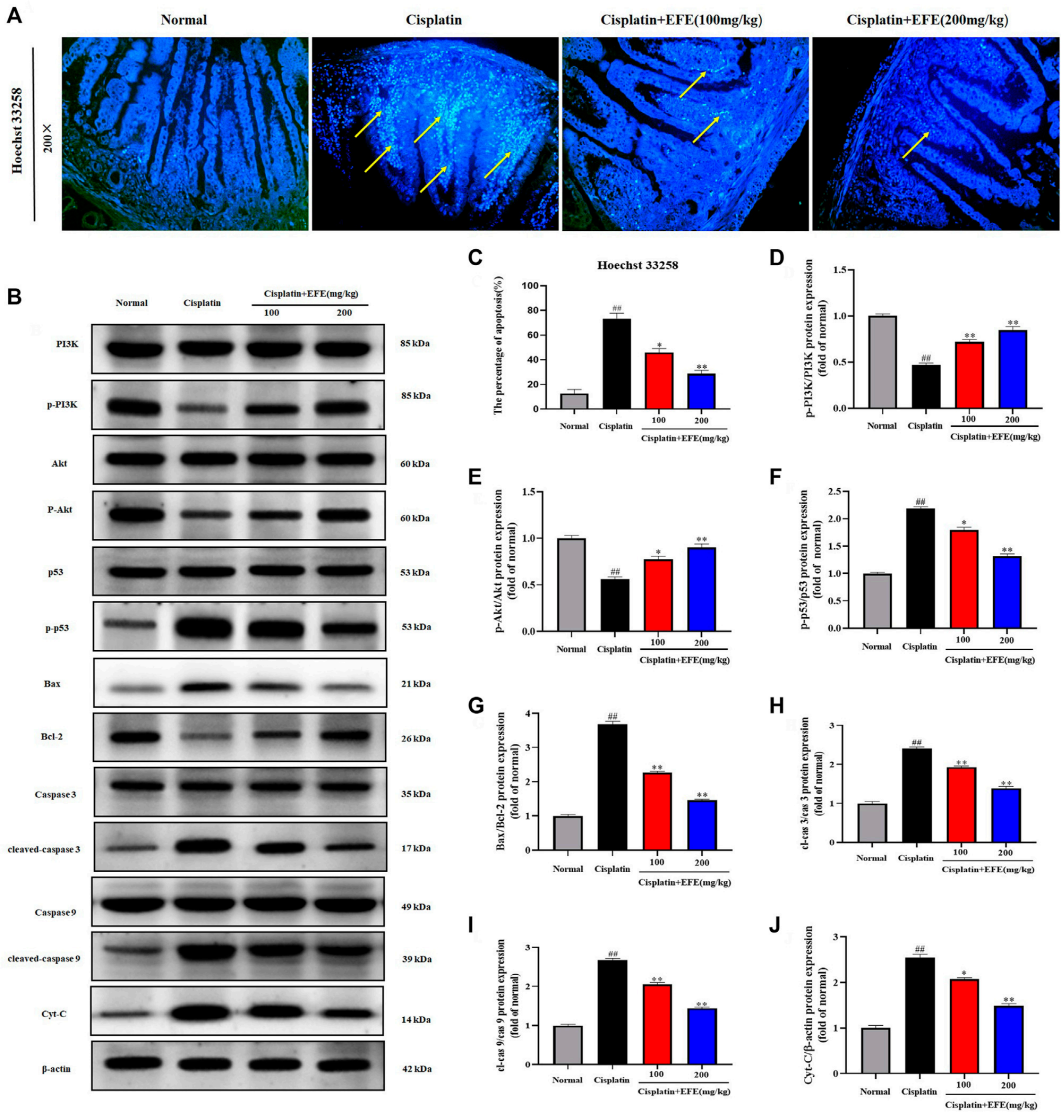
Effect of EFE on cisplatin-induced intestinal apoptosis in mice **(A)**, and the percentage of apoptosis **(C)**; Effect of EFE on p-PI3K, PI3K, p-Akt, Akt, p-p53, p53, Bax, Bcl-2, cleaved caspase-9, caspase-9, cleaved caspase-3, caspase-3, and cytochrome c expression levels in mouse intestinal tissues **(B,D–J)**. Note: Values are expressed as Mean ± S.D., (*n* = 10). ^##^ *p* < 0.01 vs. normal group; *p < 0.05, **p < 0.01 vs. model group.

As shown in Figure 6A, the intestinal histoarchitecture of normal mice was intact, neatly arranged with clear contours, and with no blue fluorescence of nuclei. Intestinal tissues of cisplatin-treated mice showed nuclear fragmentation and condensation and intense fluorescent signals, suggesting that cisplatin caused severe intestinal cell apoptosis. However, EFE could significantly reduce positive cells and improve cisplatin-induced intestinal apoptosis (Figure 6C) (*p* < 0.05 or *p* < 0.01).

To further explore the protective mechanism of EFE on cisplatin-induced intestinal apoptosis, western blotting was used to detect the expression levels of apoptosis-related proteins in mouse intestinal tissues (Figure 6B). The results showed that p-PI3K, p-Akt, and Bcl-2 proteins were significantly downregulated in the intestinal tissues of the model group, while p-p53, Bax, cleaved caspase-3, cleaved caspase-9, and cytochrome c proteins were upregulated considerably (*p* < 0.01). However, the levels of these proteins were substantially reversed after EFE administration, and especially the high-dose EFE showed better effects (Figures 6D–J) (*p* < 0.05 or *p* < 0.01).

## Discussion

Epimedii Folium is a critical Chinese herbal medicine widely used to treat various malignant diseases. According to literature reports, flavonoids extracted from Epimedii Folium could ameliorate intestinal damage by regulating multiple signaling pathways. For example, icariin, the main flavonoid in Epimedium, effectively alleviated LPS-induced impairment of intestinal goblet cell function by modulating oxidative stress, inflammation, and apoptosis (Xiong et al., 2020b). Icariin pretreatment could improve the intestinal barrier dysfunction of piglets induced by *Escherichia coli* by regulating the p38 MAPKs signal pathway (Xiong et al., 2020a). Icariin could also protect intestinal cells from hypoxia-reoxygenation (H/R)-induced oxidative stress and apoptosis by activating the SIRT1/FOXO3 signaling pathway (Zhang F. et al., 2015). Cisplatin chemotherapy has serious side effects on intestinal tissues, which dramatically affects the prognosis and quality of life of patients, and severely limits its clinical application (Zou et al., 2021). While Epimedii Folium is rich in flavonoids, and these flavonoids have been reported to antagonize the toxicity of cisplatin (Ma et al., 2015; Zhou et al., 2019).

Chinese herbal medicines show good prospects in the treatment of complex diseases owing to their fewer side effects and multi-target effects. Due to the complexity of the ingredients of traditional Chinese medicine, it is difficult to comprehensively explore the potential pharmacological activities of the drugs through the research model of animal experiments. Network pharmacology is a novel method to reveal the active components and action mechanisms of Chinese herbal medicines (Zhang Y. et al., 2015). In light of this, we employed network pharmacology and animal experiments to explore the protective mechanism of EFE on the cisplatin-induced intestinal injury. As a result, 200 drug-disease common targets were identified through database screening. Thirty key targets were selected in the hub PPI network. Molecular docking simulations indicated that the core target proteins AKT1, p53, TNF-α, NF-κB, caspase 3, and caspase 9 might play essential roles in Epimedii Folium’s treatment of intestinal injury. GO and KEGG pathway enrichment analysis suggested that the treatment of intestinal damage by Epimedii Folium involves oxidative stress, inflammation, and apoptosis.

Oxidative stress is thought to play a crucial role in the mechanism of cisplatin-induced toxicity (Hazman et al., 2018). From this perspective, there is clear evidence that flavonoid compounds with antioxidant properties can reduce cisplatin-induced toxicity (Malik et al., 2015; Arab et al., 2016; Lu et al., 2022). Cisplatin exposure disrupts the endogenous antioxidant defense system, increases the production of ROS, reduces the activity of antioxidant enzymes and causes oxidative stress (Khan et al., 2012b). ROS-mediated oxidative stress plays a vital role in the progression of cisplatin-induced intestinal dysfunction (Khan et al., 2012a). Lipid peroxidation is an important marker of oxidative stress. Cisplatin treatment significantly increased the level of MDA, a product of lipid peroxidation in tissues (Karadeniz et al., 2011). Studies have reported that the activities of antioxidant enzymes GSH-Px and CAT in intestinal tissue of cisplatin-treated rats decreased, and the level of MDA increased (Shahid et al., 2017; Hu et al., 2021). Epimedii Folium, as a natural antioxidant, has been reported to protect the antioxidant defense system by increasing the activity of antioxidant enzymes GSH-Px, CAT, and SOD while reducing the content of lipid peroxidation marker MDA (Yang et al., 2020; Zhao et al., 2022). Our results were consistent with literature reports, suggesting that EFE could exert an excellent protective role against cisplatin-induced intestinal injury by resisting oxidative stress.

Oxidative stress and inflammation are closely related in biological systems (Araujo et al., 2017). In addition to direct toxicity to the body, ROS can also induce the expression of the nuclear transcription factor NF-κB, thereby increasing the production of cell adhesion molecules, chemokines, and proinflammatory cytokines, and further enhancing the cytotoxicity of cisplatin (Li et al., 2017). Transcription of inflammatory markers such as iNOS, COX-2, TNF-α, and IL-1β can be triggered explicitly by activation of NF-κB (Liu et al., 2021). According to literature reports, Epimedii Folium can significantly reduce the expression of nuclear transcription factor NF-κB and the secretion of proinflammatory cytokines TNF-α and IL-1β (Huang et al., 2018; Yan et al., 2018). Our findings support this conclusion, showing that EFE can attenuate cisplatin-induced intestinal injury by inhibiting inflammation.

Previous studies have demonstrated that cisplatin-induced toxicity is closely related to oxidative stress-induced apoptosis (Shahid et al., 2018). When cisplatin induces ROS overproduction, it can cause mitochondrial dysfunction, change mitochondrial permeability, trigger the opening of the mitochondrial permeability transition pore (MPTP), release cytochrome c from mitochondria to the cytoplasm, and lead to the successive activation of caspase 9 and its downstream caspase 3, thereby inducing a mitochondrial-dependent pathway and ultimately leading to apoptosis (Kim et al., 2003). Cisplatin-induced intestinal toxicity mechanisms include oxidative stress, p53, and apoptosis through upregulation of caspase-3 and caspase-6 expression (Khan et al., 2012a). Bax and Bcl-2 are the most representative pro-apoptotic and anti-apoptotic factors. Cisplatin treatment significantly increased the expression of Bax and inhibited the expression of Bcl-2. Studies have shown that flavonoids extracted from Epimedii Folium can exert anti-apoptotic effects by increasing the ratio of Bcl-2/Bax and reducing the expression of caspase 3 (Liu et al., 2014; Hu and Ma, 2021). Our results also showed that EFE alleviated cisplatin-induced apoptosis by downregulating Bax expression, upregulating Bcl-2 expression, reducing caspase-9 and caspase-3 expression, and cytochrome c release.

PI3K-Akt is a crucial intracellular signal transduction pathway that plays a crucial role in cell growth, proliferation, and migration (Zhang et al., 2020). It is also a survival signaling pathway that can resist various apoptotic injuries (Qin et al., 2021). Network pharmacology and molecular docking results suggested that the PI3K-Akt signaling pathway was the principal pathway of Epimedii Folium against intestinal damage. Furthermore, given the regulatory effects of Epimedii Folium on cell proliferation and the PI3K/Akt pathway (Song et al., 2014), this study validated the role of the PI3K-Akt pathway in the treatment of cisplatin-induced intestinal injury by EFE through western blotting. Excitingly, we demonstrated that EFE could significantly ameliorate cisplatin-induced intestinal injury by modulating the PI3K/Akt pathway.

Mitochondrial DNA is one of the main targets of cisplatin (Coskun et al., 2014). Cisplatin forms covalent adducts between platinum atoms and DNA bases, causing irreparable damage to DNA, inhibiting the synthesis of nucleic acids and proteins, and leading to apoptosis (Sancho-Martinez et al., 2012). Cisplatin-induced cell death is more intense in mitochondria-rich duodenal epithelial cells (Qian et al., 2005). p53, a tumor suppressor protein, is a crucial mediator of the DNA damage response and is considered to play a vital role in cisplatin-induced toxicity (Jiang and Dong, 2008). After cisplatin treatment, the p53 protein is activated and transferred to mitochondria, leading to the activation of the caspase pathway, which ultimately induces apoptosis (Khan et al., 2012b). EFE could inhibit cisplatin-induced apoptosis by reducing the expression of the p-p53 protein.

## Conclusion

**FIGURE 7:**
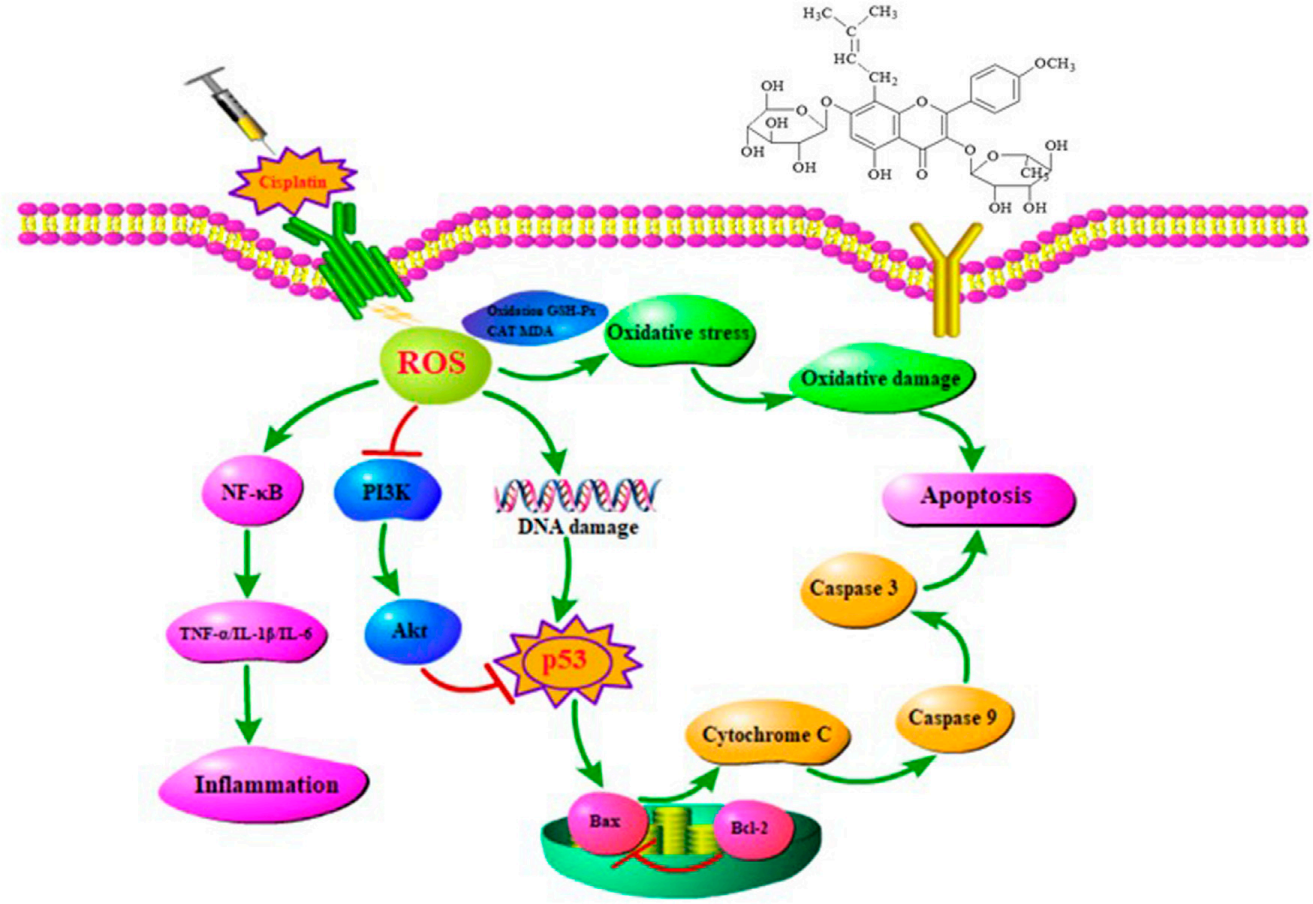
The mechanism of EFE attenuates cisplatin-induced intestinal injury in mice.

In conclusion, combing with network pharmacology, molecular docking, and animal experiments, our study demonstrated for the first time that EFE could significantly attenuate cisplatin-induced intestinal damage by regulating oxidative stress, inflammation, and apoptosis. The molecular mechanism of action might be mainly related to PI3K/Akt, p53, and NF-κB signaling pathways (Figure 7). These findings could provide a theoretical basis for the clinical treatment of the toxic and side effects of cisplatin chemotherapy, guide the application of antitumor drugs, and provide new ideas for the application and development of Epimedii Folium in the future.

## Data Availability

The original contributions presented in the study are included in the article/supplementary materials, further inquiries can be directed to the corresponding authors.

## Abbreviations

EFE, Epimedii Folium extract; cTnT, Plasma cardiac troponin T; TNF-α, Tumor necrosis factor-α; GSH-Px, Glutathione peroxidase; MDA, Malondialdehyde; PI3K, Phosphatidylinositol 3-kinase; NF-κB, Nuclear factor-kappa B; CK-MB, Creatine kinase isoenzyme MB; LDH, Lactate dehydrogenase; IL-1β, Interleukin-1β; CAT, Catalase; IL-6, Interleukin-6; Akt, Protein kinase B; Cyt-C, Cytochrome C
